# Do Women With High eHealth Literacy Profit More From a Decision Aid on Mammography Screening? Testing the Moderation Effect of the eHEALS in a Randomized Controlled Trial

**DOI:** 10.3389/fpubh.2019.00046

**Published:** 2019-03-12

**Authors:** Maren Reder, Renate Soellner, Petra Kolip

**Affiliations:** ^1^School of Public Health, Bielefeld University, Bielefeld, Germany; ^2^Institute for Psychology, Department of Education and Social Sciences, University of Hildesheim, Hildesheim, Germany

**Keywords:** eHealth literacy, eHEALS, decision aid, mammography screening, knowledge

## Abstract

**Background:** Our decision aid on mammography screening developed according to the criteria of the International Patient Decision Aids Standards Collaboration increases knowledge compared to usual care. However, it remains unclear whether this decision aid is more effective in women with higher eHealth literacy. Our objective was to test whether the positive effect of the decision aid on knowledge is moderated by eHealth literacy.

**Methods:** A total of 1,206 women aged 50 from Westphalia-Lippe, Germany, participated (response rate of 16.3%) in our study and were randomized to usual care (i.e., the standard information brochure sent with the programme's invitation letter) or the decision aid. eHealth literacy was assessed at baseline with the Electronic Health Literacy Scale (eHEALS); knowledge was assessed at baseline and post-intervention. First, we compared the 2-factor model of the German eHEALS (information-seeking and information-appraisal) found in previous research and the 3-factor model we hypothesized for decision aid use to the originally proposed 1-factor model. Second, we modeled the measurement model according to the superior factor model found in step one and tested whether the eHEALS moderated the effect of the decision aid on knowledge.

**Results:** The 3-factor model of the eHEALS had a better model fit than the 1-factor or 2-factor model. Both information-seeking, information-appraisal, and information-use had no effect on knowledge post-intervention. All three interactions of the decision aid with information-seeking, information-appraisal, and information-use were not significant. Equally, neither education nor its interaction with the decision aid had an effect on knowledge post-intervention.

**Conclusion:** The decision aid developed in this project increases knowledge irrespective of level of eHealth literacy. This means that not only women with high eHealth literacy profit from the decision aid but that the decision aid has been successfully conceptualized as a comprehensible information tool that can be used by women of varying eHealth literacy levels.

**Trial registration**: German Clinical Trials Register DRKS00005176 (https://www.drks.de/drks_web/navigate.do?navigationId=trial.HTML&TRIAL_ID=DRKS00005176).

## 1. Introduction

In various health domains, decision aids (DAs) increase knowledge (1). One of these domains is mammography screening, for which increasing knowledge is of special importance due to widespread overestimation of benefits [e.g., more than 90 % overestimate the reduction in breast cancer mortality (2)] and it being unclear whether benefits outweigh harms (3). For women aged 40 and women aged 70 deciding about mammography screening participation, positive effects of a DA on knowledge have been reported (4, 5). However, neither of these DAs is aimed at the target group of mammography screening: women aged 50–69. For the context of the Mammography Screening Programme in Germany (MSP), we developed a DA according to the International Patient Decision Aid Standards (IPDAS) (6) for women aged 50 who are invited to the MSP for the first time. Similar to previous research, this DA increased knowledge about mammography screening compared to usual care at both post-intervention and 3-month follow-up (7).

Our DA follows a one-size-fits-all approach whereas women using the DA differ on many dimensions. Some of the relevant dimensions may be health literacy, preferred language, existing knowledge levels, and differing motivations regarding engagement with the decision (7). Research shows that effectively using a DA may be influenced by the user's health literacy (8). In a systematic review of DAs, lower health literacy was associated with lower health knowledge (9). People with lower health literacy are less likely to benefit from health education materials because they have difficulty comprehending written information making them less likely to acquire new knowledge (10). For example, lower performance on the Rapid Estimate of Adult Literacy in Medicine (REALM) was associated with lower mammography knowledge (11). Importantly, women with low health literacy also lack the numeracy skills to understand the likelihood of benefits and harms of mammography screening (12). Therefore, it is questionable whether our one-size-fits-all approach is adequate regarding different levels of health literacy of the recipients. Women with a lower level of health literacy could benefit less from our DA making a targeted approach for this group necessary.

Many conceptual approaches to health literacy exist (13, 14) including the construct of eHealth literacy. Health literacy can be defined as a set of cognitive, social, and motivational skills that enable people to access, understand, and use information for health (15). eHealth literacy can be defined as ability to seek and appraise information in electronic sources and to use this information (16). Both constructs (health literacy and eHealth literacy) overlap considerably and their definitions mainly differ by the mode health information is consumed (through the Internet or through traditional modes) (17). Since our DA was an online interactive tool, we assumed that eHealth literacy was the more adequate construct. Thus, we assumed eHealth literacy to better envelop the skills for successful DA use than traditional health literacy. To assess eHealth literacy, we used the eHealth literacy scale (eHEALS) (18). It measures the perceived ability to find, evaluate and use health related information gained in electronic environments (18) and is the most used tool for eHealth literacy assessment (19). We assumed knowledge to be the most likely outcome to be influenced by eHealth literacy because knowledge has been reported in previous research to be strongly affected by both decision aids (1) and eHealth literacy (9). For both attitude and intention/uptake (i.e., the other two dimensions of informed choice), neither did previous research (1) nor our results (7) show consistent effects of DAs. Testing a moderation of eHealth literacy on these outcomes was therefore not indicated.

The factor structure of the eHEALS is somewhat controversial. The eHEALS was originally developed as a 1-dimensional scale, which was confirmed through principal component analysis in a randomized intervention trial with secondary school students in Canada (18). The Dutch (20), the Japanese (21), the Chinese (22), and the Italian (23) versions of the eHEALS all showed a 1-factor structure even though they were assessed in very different samples. Additionally, in a sample of people aged 50 and older in the U.S.A., a 1-factor structure was found (24). Contrastingly, in a previous study among 327 18-year-old students, we showed that the German version of the eHEALS consists of two factors: information-seeking and information-appraisal (14). Six of the eHEALS items (information-seeking subscale) either focus on the ability to find information on the Internet (I 1, I 3, I 4) or on the ability to use this information for health decisions (I 2, I 5, I 8) (14). The other Items (I 6, I 7; information-appraisal subscale) cover the ability to evaluate information sources (14). For using the DA, we assumed it to be most important to have the ability to use information for health decisions. We therefore decided to additionally test a 3-factor solution in which the information-seeking subscale was split in two 3-item subscales: information-seeking (I 1, I 3, I 4) and information-use (I 2, I 5, I 8). Thus, our objectives were to (1) test the factor structure of the eHEALS and to (2) test whether the effect of the decision aid on knowledge is moderated by eHealth literacy. In the second step eHealth literacy was modeled according to results of step 1.

## 2. Materials and Methods

The protocol [for details see the published study protocol (25) and the CONSORT checklist provided as supplementary material of a previous publication (7)] for this two-armed RCT was approved by the Ethics Commission of the Medical Association Westphalia-Lippe and the Medical Faculty of the University of Münster. All participants gave written informed consent. This RCT has been registered in the German Clinical Trials Register under trial-ID DRKS00005176 (https://www.drks.de/drks_web/navigate.do?navigationId=trial.HTML&TRIAL_ID=DRKS00005176). Blinding was disregarded because it was obvious for the women whether they received a DA.

### 2.1. Participants and Procedure

Women aged 50 (i.e., first time invitees to the MSP) were eligible for this study. Not eligible were women with potential Turkish migration background due to simultaneous recruitment for another study focussing on this group (26). Data (name and address) on the population of women of the birth months March to May 1964 with residence in Westphalia-Lippe, North Rhine-Westphalia, Germany was provided by registration offices. Of these women, we randomly drew a sample of 7,400 women.

A total of 1,206 women aged 50 participated (response rate of 16.3%) in our study and were randomized to usual care (i.e., the standard information brochure (27) sent with the programme's invitation letter) or the DA (i.e., they received the usual care brochure and the DA). The standard information brochure comprised written and numerical information about the MSP (7, 25). Women were informed about their study group at the second assessment (when they received the link to the DA).

The study invitation was mailed 3 weeks prior to the estimated arrival of the MSP invitation. Three weeks after the study invitation, the link to the baseline questionnaire was e-mailed to the participating women. Assessments were conducted at baseline, post-intervention (2 weeks after baseline), and 3 months follow-up (7). Data were collected between April and November 2014. Data from all measurement points were linked through self-generated codes.

### 2.2. Decision Aid

We developed a DA for women invited to the MSP for the first time based on the criteria of the International Patient Decision Aids Standards Collaboration [see the BARMER website where our DA (the DA is in German) was made available after the end of our study (https://www.barmer.de/gesundheit/praevention/krebspraevention/krebsfrueherkennung/mammographie-13876) and the Decision Aid Library Inventory where it was registered (https://decisionaid.ohri.ca/AZsumm.php?ID=1673)] (7). The DA consisted of a static information part and an interactive part. Its structure was based on Mathieu et al.'s DA (5). The chance of each outcome was expressed using absolute numbers. These numbers were illustrated by crowd-figure-pictograms (200 women over 20 years) on breast cancer mortality with and without mammography screening, false positives, breast cancer diagnoses, and interval cancers (7). The information from the brochure in the MSP invitation (27) was included in our DA. The interactive part of the DA encouraged engagement with the information: Assigning the information items to the categories “in favor of mammography screening,” “neither for nor against such screening,” or “against the screening,” rating the importance of each information item, and making a choice (7). At the end, women received a tailored summary. For a sample of the tailored summary including the crowd-figure-pictograms, see the supplementary material of our previously published study protocol (25).

The DA and the usual care brochure thus, differed on three key aspects: (1) The DA contained an interactive part with three steps (assigning the information items to categories, rating the importance of each information item, making a choice) displaying a graphical summary of personal responses at the end (7). (2) In the information part of the DA, absolute numbers were illustrated by crowd-figure-pictograms (200 women over 20 years) (7). (3) We included information on all cause mortality (7).

### 2.3. Outcome Measures

The questionnaires were based on the questionnaire of the InEMa study (28) and adapted for the evaluation of an randomized controlled trial. Education was assessed with one question comprising the following answer options (German degrees are given followed by years of education): “Hauptschulabschluss” (9 years of education), “Realschulabschluss” (10 years of education), “Polytechnische Oberschule” (degree awarded in the former German Democratic Republic, 10 years of education), “Fachhochschulreife” (11 to 12 years of education, qualification for attendance of universities of applied sciences), “Abitur” (12 to 13 years of education, qualification for attendance of universities), other, and no school degree. For the analysis, we dichotomized these different degrees into low education (degrees with up to 10 years of education) and high education (degrees with more than 10 years of education).

#### 2.3.1. Knowledge

Knowledge was assessed at baseline and post-intervention using seven multiple choice items on (1) target group of the MSP, (2) number of women receiving a positive result, (3) whether a positive screening result equals a diagnosis, (4) existence of false negatives, (5) number of diagnoses in screened vs. unscreened populations, (6) number of breast cancer deaths in screened vs. unscreened populations, and (7) existence of overtreatment (7, 25). All questions, except Question 2 which assessed numerical knowledge on the number of women receiving a positive result in number categories (1 to 20 of 200, 21 to 50 of 200, 51 to 100 of 200, 101 to 200 of 200), assessed conceptual knowledge (7, 25).

#### 2.3.2. eHealth Literacy

eHealth literacy was assessed at baseline using the German translation (14) of the eHEALS (18). It comprises eight items covering (1) knowing how to find information online, (2) knowing how to use the internet to answer questions, (3) knowing what health resources are available, (4) knowing where to find health resources, (5) knowing how to use this health information, (6) having the skills to evaluate health resources, (7) ability to discriminate between high and low quality resources, and (8) confidence to use information to make health decisions. Responses were given on a 5-point Likert scale (1 = strongly disagree, 5 = strongly agree).

### 2.4. Statistical Analysis

Data were analyzed with SPSS version 24 (IBM, Corp., Armonk, NY) and MPlus version 8 (Muthén & Muthén, Los Angeles, CA). In a first step, we compared the 2-factor model of the eHEALS we found in previous research on 18-year-old students (14) to the originally proposed 1-factor model (18). Then we compared our proposed 3-factor-model with the best-fitting-model.

In a second step, we modeled eHealth literacy according to the superior factor model found in step one and tested whether this moderated the effect of the DA on knowledge. For both steps, latent structural equation models were used. Both, knowledge and eHealth literacy were modeled as latent variables which allowed (1) to account for measurement error, (2) to test measurement invariance, and (3) to apply full information maximum likelihood estimation enabling us to include individuals with missing values in the analysis (7). All models were calculated using the Fixed-Factor-Method (29).

Two types of latent analyses were conducted. For step 1, the numeric eHEALS items forming one or more latent factors were analyzed using confirmatory factor analysis (CFA). CFAs of the 1-, 2-, and 3-factor models were conducted. Model fit was compared using χ^2^-tests (30). Additionally, the following model fit indices were assessed to compare the fit of the models: Comparative Fit Index (CFI) > 0.95, Tucker-Lewis Index (TLI) > 0.95, Root Mean Squared Error of Approximation (RMSEA) < .06, Standardized Root Mean Squared Residual (SRMR) < 0.08 (31), Akaike Information Criterion (AIC) as small as possible, and Bayesian Information Criterion (BIC) as small as possible (32).

The latent analysis for the second step modeled a first order autoregressive effect of knowledge at T1 on knowledge at T2 in concordance with our previous analyses of knowledge (7) (i.e., we used the same autoregressive model for testing the intervention effect (DA vs. usual care) on knowledge). The categorical knowledge items forming a latent factor were analyzed using 2-parameter-logistic item factor analysis. Model fit information for these models is somewhat limited since the maximum likelihood estimation only provides indices of relative model fit (loglikelihood value, AIC, BIC). For the loglikelihood value, a larger value (indicating the maximization of the loglikelihood function) is better (33). For these models, the assumption of invariance held, if the loglikelihood difference test was not significant (34, 35). This had already been established in previous research (7). With these 2 measurement models for our two latent constructs, the structural model estimated the effect of knowledge at T1, the DA, the components of the eHEALS and their interactions with the DA on knowledge at T2.

## 3. Results

### 3.1. Sample Characteristics

Code matching of all measurement points provided 1,052 datasets. Women who ever had breast cancer (*n* = 29) or did not respond to this question (*n* = 26) or self-reported at T2 that the MSP appointment had passed (*n* = 84) were excluded (7). Accordingly, 913 women were included in the analyses. Background and outcome variables were similar between groups (7). Nearly 60 % had already received the invitation to the MSP and the associated brochure at baseline (7). Most women in our sample had an intermediate school certificate (41.2%), followed by a university entrance qualification (30.8%), a university of applied sciences entrance qualification (14.7%), a secondary general school certificate (10.4%), and other/no degrees (2.9%). Thus, the majority of women had a school education of up to 10 years (control: 53.0%, DA: 55.2%). 47.0% (control) and 44.8% (DA) had a school education of more than 10 years.

One third of women spent 1–2 h per week searching for information on the Internet (control: 34.5%, DA: 31.9%), another third spent 2–5 h (control: 33.0%, DA: 30.3%). Few women spent <1 h (control: 16.0%, DA: 18.1%). The Internet as information source for health topics was rated as important by the majority (control: 58.3%, DA: 57.8%). Few women rated the Internet as unimportant (control: 14.6%, DA: 18.0%). For further information on the baseline characteristics of the sample see Table 2 in (7).

The response frequencies to the eHEALS items are shown in Table 1. For items 1, 2, 4, 5, and 6, the most frequent response was “agree.” For Items 3, 7, and 8, the most frequent response was “neutral.” Nevertheless, even for those items (except item 8), more women agreed with the item than disagreed. Item 8 was the only item where more women disagreed than agreed. This may be because this is the most advanced item (using information to make actual health decisions). Looking at the category “strongly agree,” this pattern repeats itself; for all other items, between eleven and thirty per cent chose this category while for Item 8 only 3% chose this category.

**Table 1:** Response frequencies for all eHEALS items.

**Number**	**Item**	**Strongly disagree**	**Disagree**	**Neutral**	**Agree**	**Strongly agree**
1	I know how to find helpful health resources on the Internet	20 (2.3)	59 (6.7)	282 (31.9)	362 (41.0)	161 (18.2)
2	I know how to use the Internet to answer my questions about health	28 (3.2)	66 (7.5)	226 (25.5)	369 (41.7)	196 (22.1)
3	I know what health resources are available on the Internet	32 (3.6)	104 (11.8)	356 (40.2)	267 (30.2)	126 (14.2)
4	I know where to find helpful health resources on the Internet	18 (2.0)	83 (9.4)	314 (35.6)	319 (36.2)	148 (16.8)
5	I know how to use the health information I find on the Internet to help me	22 (2.5)	59 (6.7)	295 (33.3)	351 (39.7)	158 (17.9)
6	I have the skills I need to evaluate the health resources I find on the Internet	12 (1.4)	38 (4.3)	165 (18.6)	402 (45.4)	268 (30.3)
7	I can tell high quality health resources from low quality health resources on the Internet	40 (4.5)	109 (12.3)	355 (40.0)	286 (32.2)	98 (11.0)
8	I feel confident in using information from the Internet to make health decisions	79 (8.9)	196 (22.1)	446 (50.4)	135 (15.3)	29 (3.3)

*Data are given as number (percentage)*.

The proportion of women with adequate knowledge at T1 was less than one-third (control: 29.8%, DA: 28.6%) (7). At T2, 66.8% had adequate knowledge in the DA group and 31.4% in the control group (7). The 2-parameter-logistic item factor analysis on knowledge (7) showed that item 6 (Who is more likely to die of breast cancer? Women participating in the MSP/ Women not participating in the MSP/ Both the same) had negative loadings. Therefore, this item was excluded from all further analyses (7). Knowledge has already been shown to be partially strong measurement invariant over all three measurement points (7).

### 3.2. Factor Structure of the eHEALS

**Figure 1:**
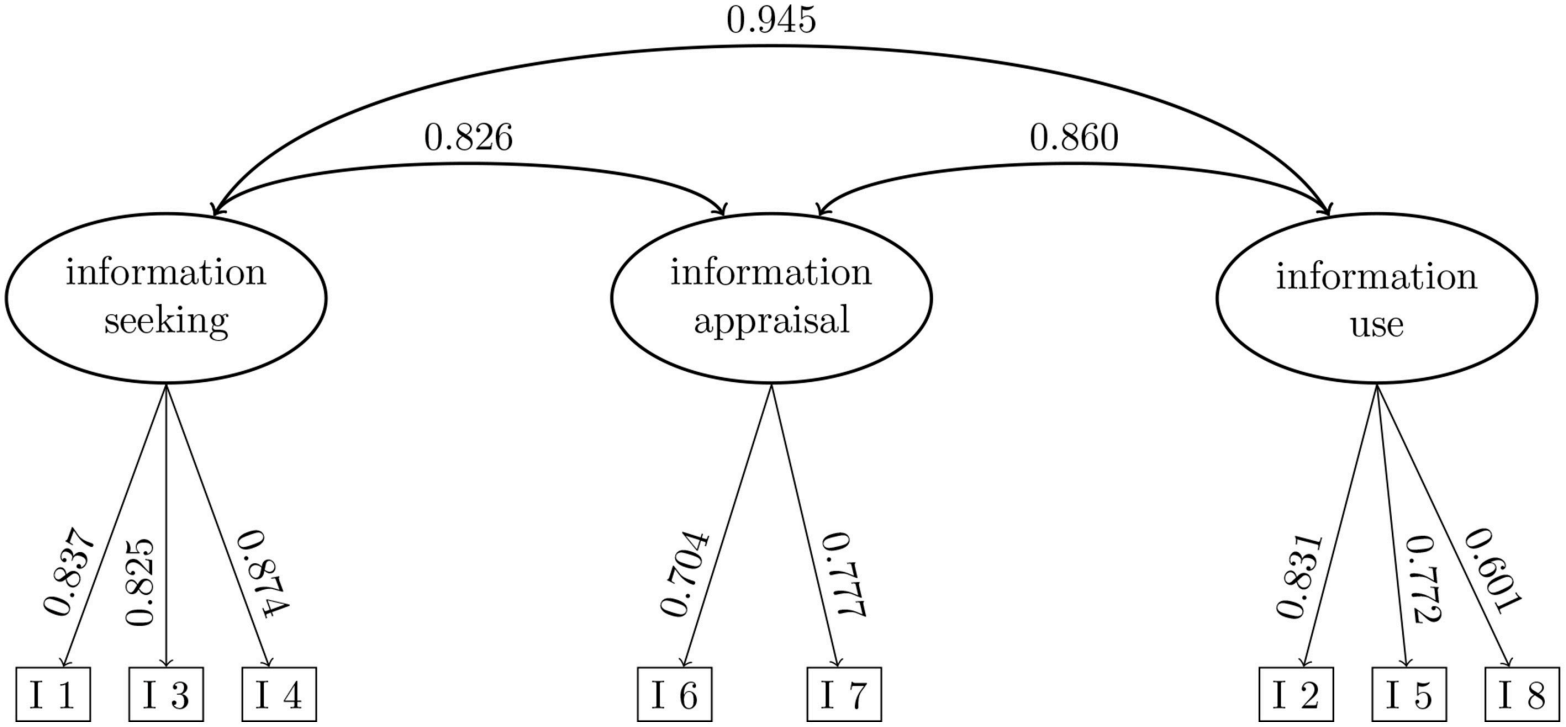
3-factor model of the eHEALS. I 1 to I 8 refer to the item numbers.

The 2-factor model of the eHEALS had a better model fit than the 1-factor model; the 3-factor model (see Figure 1) had a better model fit than the 2-factor model. All model fit indices are depicted in Table 2. Both χ^2^ differences were significant (Model 1 vs. Model 2: χ^2^ = 50.64 (1); *p* < 0.001; Model 2 vs. Model 3: χ^2^ = 29.04 (2); *p* < 0.001). The CFI, TLI, SRMR, RMSEA, AIC, and BIC indicated the best model fit for the 3-factor model. Nevertheless, the CFI and TLI and the SRMR of the other models indicated good model fit. Only the RMSEA was > 0.06 for Models 1 and 2. For the 3-factor model, the covariance between information-seeking and information-appraisal was 0.83, between information-appraisal and information-use 0.86, and 0.95 between information-seeking and information-use.

**Table 2:** Confirmatory factor analysis of the three models of the eHEALS.

**Model**	**Chi-Square (df)**	**CFI**	**TLI**	**RMSEA**	**SRMR**	**AIC**	**BIC**
Model I Factor 1: 1-8	147.52 (20)	0.968	0.956	0.085	0.029	15417.666	15532.683
Model II Factor 1: 1-5, 8 Factor 2: 6, 7	96.88 (19)	0.981	0.971	0.068	0.022	15369.025	15488.833
Model III Factor 1: 1, 3, 4 Factor 2: 6, 7 Factor 3: 2, 5, 8	67.84 (17)	0.987	0.979	0.058	0.019	15343.986	15473.380

*Chi-Square Difference between Model I and Model II: χ^2^ = 50.64 (1); p < 0.001. Chi-Square Difference between Model II and Model III: χ^2^ = 29.04 (2); p < 0.001*.

### 3.3. Interaction Between the Decision Aid and eHealth Literacy

**Figure 2:**
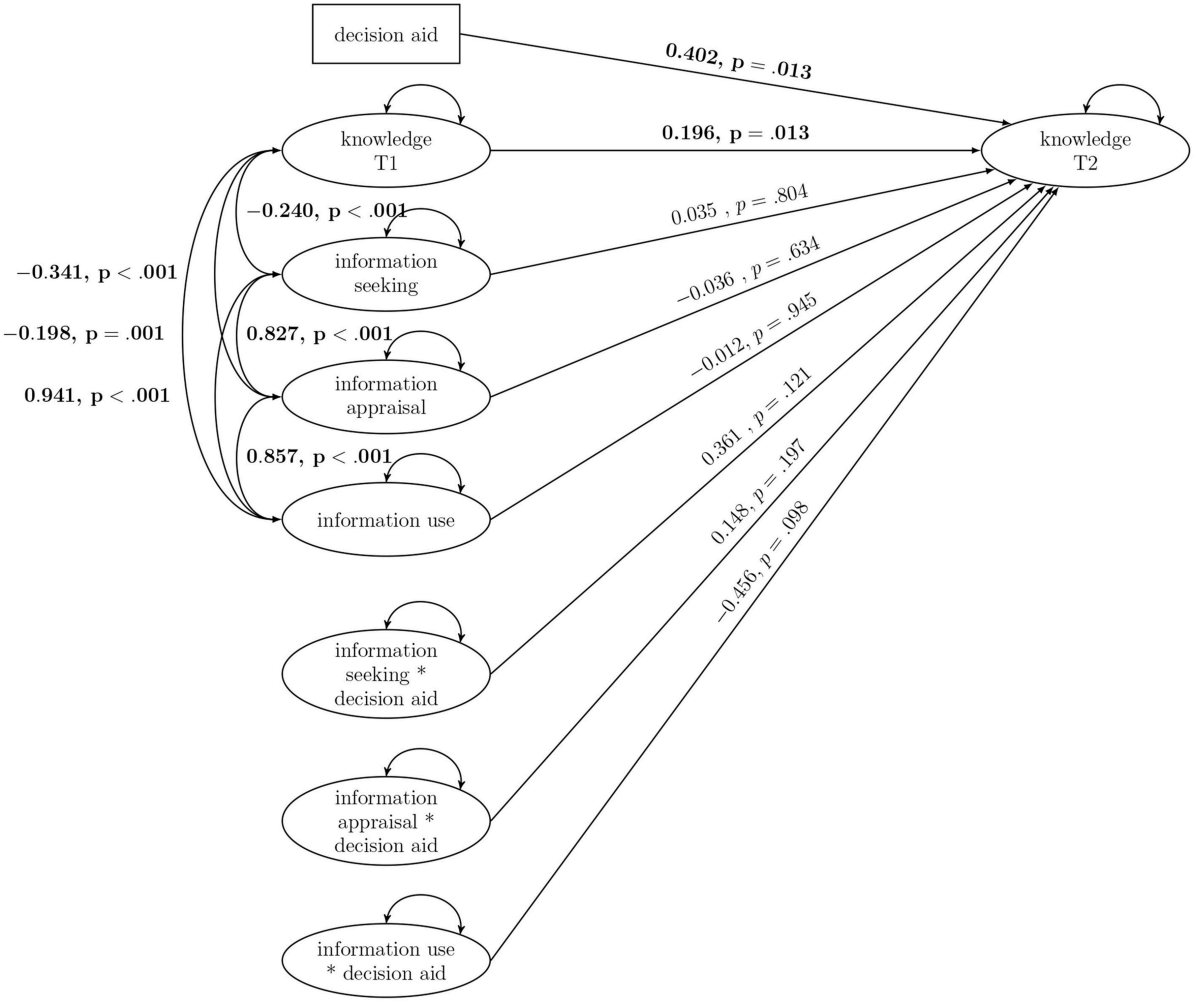
Interaction model of eHealth literacy and the decision aid. Significant results are bolded.

The interaction model showed the following model fit: Loglikelihood = −12297.036, df = 69, AIC = 24732.072, BIC = 25063.514. The DA significantly increased knowledge at T2 (see Figure 2). Information-seeking, information-appraisal, and information-use had no effect on knowledge at T2 in the autoregressive model in which knowledge at T1 predicted knowledge at T2 (β = 0.196, *p* = 0.013). The interactions of the DA with information-seeking, information-appraisal, and information-use were not significant. Thus, the hypothesized moderation effect of information-use on the effect of the DA was not confirmed. Information-seeking, information-appraisal, and information-use showed high positive covariances (0.857 to 0.941). Contrary to our assumptions, information-seeking, information-appraisal, and information-use all showed negative covariances with knowledge at T1 (−0.198 to −0.341). This means that people with higher levels of information-seeking, -appraisal, and -use had lower levels of knowledge at T1 and vice versa.

**Figure 3:**
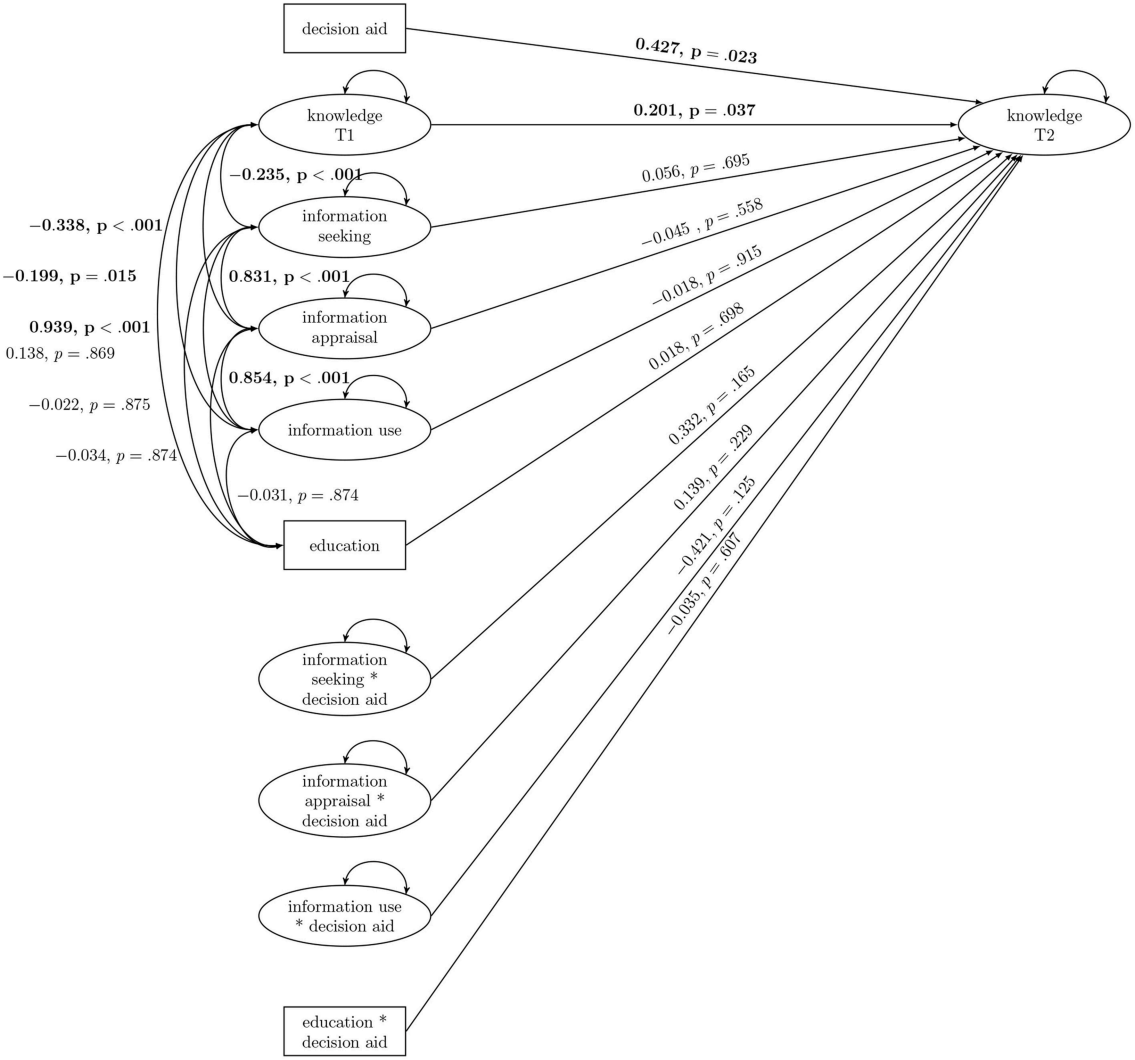
Interaction model of eHealth literacy, education, and the decision aid. Significant results are bolded.

In a second model, we included education (low vs. high) and its interaction with the DA (see Figure 3). This model showed the following model fit: Loglikelihood = −12723.310, df = 77, AIC = 25600.619, BIC = 25968.848. Again, the DA and knowledge at T1 had a significant effect on knowledge at T2. All eHEALS subscales as well as their interactions with the DA remained non-significant. Education and its interaction with the DA both did not predict knowledge at T2. Education was not significantly associated with any of the other outcomes measured at T1. Information-seeking, information-appraisal, and information-use were again all significantly and positively associated. As in the previous model, knowledge at T1 was associated significantly and negatively with all eHEALS subscales.

## 4. Discussion

Our objectives were to (1) test the factor structure of the eHEALS and to (2) test whether the effect of the DA on knowledge was moderated by eHealth literacy. For the factor structure of the eHEALS, the 3-factor model (information-seeking, information-appraisal, and information-use) showed the most favorable model fit. All three subscales had no effect on knowledge at T2. The effect of the DA on knowledge was not moderated by any eHealth literacy subscale. Similarly, education and its interaction with the DA did not predict knowledge at T2.

Regarding the factor structure of the eHEALS, the 3-factor model showed the most favorable model fit. Nevertheless, we could confirm the superiority of the 2-factor model over the 1-factor model, which has been shown in previous research for the German translation in 18-year-old high school students (14) where the eHEALS items 1 to 5, and 8 measured information-seeking and items 6 and 7 assessed information-appraisal. Neter et al. conducted a CFA of the Hebrew translation of the eHEALS (36) and both the previously proposed 1- and 2-factor models showed poor fit (37). A subsequent EFA followed by a CFA in the second half of the sample yielded a good-fitting alternative 2-factor model still measuring information-seeking and information-appraisal (37). Additionally, Richtering et al. reported their PCA to suggest two factors (in this research, items 1 to 5 assessed knowledge about resources and items 6 to 8 assessed evaluation of resources) (38). Diviani et al. (39) conducted a CFA of the Italian version of eHEALS indicating that both the 1- [as originally proposed (18)] and 2-factor model developed by Soellner et al. (14) showed inadequate model fit although the 2-factor model had better model fit than the 1-factor model. Contrastingly, their item response theory analyses indicated a 1-factor model (39).

In line with our results that the 3-factor model fitted best, Stellefson et al. comparing a 1-, 2- and 3-factor model of the eHEALS with exploratory structural equation modeling found a 3-factor solution to show the best model fit (40). Similarly, Sudbury-Riley et al. (41) testing a 3-factor structure with CFA in a three country sample (UK, US, New Zealand) reported that the eHEALS comprised 3-factors. It has to be noted that these factors were different from the three factors we found (items 1 and 2 assessed awareness of internet health resources, items 3 to 5 assessed skills to access internet health resources, and items 6 to 8 assessed the belief in ones ability to evaluate internet health resources). Hyde et al. were able to replicate this 3-factor structure (42).

Even though there are findings pointing at a 2- or 3-factor structure of the eHEALS, most previous research indicated that the eHEALS is unidimensional. Regardless of the empirical findings, the theoretical arguments for unidimensionality or multidimensionality have received too little attention in the past (41). Most studies used PCA (18, 20, 22, 36) even though CFA provides more rigorous results (43). This may indicate that the theoretical dimensionality of eHealth literacy has been neglected resulting in theoretically unfounded interpretations of the eHEALS's factorial structure (42). The eHEALS has been translated and its factorial structure has been assessed in Dutch (20), Japanese (21), Chinese (22), German (14), Spanish (44), Italian (23), Iranian (45), and Hebrew (36, 37). Research employing PCA, almost exclusively indicated unidimensionality while studies using CFA or IRT analysis showed mixed results regarding the factorial structure.

The eHEALS is theoretically grounded on a multidimensional model but Norman and Skinner postulated their scale to be unidimensional (18). Our research results give evidence that a multi-factor structure fits the data better, as has previous research. A multi-factor structure based on theoretical assumptions of the researchers can only be evaluated by applying CFAs—more precisely CFA difference tests. For most of the previous research, we have to conclude that it remains unknown which results CFA difference test would have rendered. Regardless of this point which will have to be elucidated in future research, it has to be noted that in our model all three subscales showed high positive covariances indicating that they measure distinct but related constructs.

eHealth literacy neither had an effect on knowledge at T2 nor did it moderate the effect of the DA on knowledge. This means that the DA developed in this project increases knowledge irrespective of the level of eHealth literacy women have. This indicates that the DA has been successfully conceptualized as a comprehensible information tool that can be used by women of varying eHealth literacy levels. Other crossectional research employing adjusted analyses indicated that self-reported health literacy has no association with perceptions about colon cancer (risk of diagnosis, risk of death, benefit of screening) (46). Similar to our results though using a tailored intervention, Paasche-Orlow et al. reported that health literacy did not affect knowledge post-intervention (47). The authors concluded that low health literacy is not necessarily a barrier to profiting from health interventions and gaining knowledge (47)—at least when they are tailored.

Contrastingly, a review indicated that there is a significant and positive association between literacy and knowledge about health services and health outcomes (9). Notably, most studies in this review used skill-based measures of health literacy (9) and accordingly the results may only be compared to our results with caution. Low health literacy was associated with inadequate understanding of prenatal screening tests (48). Another review suggests that low health literacy individuals are less able to benefit from DAs - at least as long as health literacy is not sufficiently taken into consideration during the design phase (8). It may be that we did not find any effects of eHealth literacy on knowledge because our DA has been so designed as to fulfil these recommendations (8) that have been shown to support comprehension. Essential information was presented first (49). Numerical information was presented in crowd-figure-pictograms (50, 51), with the same denominator (51), and using natural frequencies (50).

Another possible explanation—at least for not finding an interaction with the subscales information-seeking and information-appraisal—is that since our respondents did not have to find the DA or appraise it, these facets may not have been relevant for successfully using the DA. Information-seeking is an important real life skill but in our study, women were provided with a link to the DA and therefore, did not have to engage in information-seeking. Similarly, it can be argued that since the DA came from a reliable source (University research project that passed through an ethics committee), the source may not have needed much appraisal skills.

Education and its interaction with the DA did not predict knowledge at T2. In previous research, knowledge about mammography screening was lower among women with low education levels (28). Less than 12 years of education were also associated with inadequate understanding of prenatal screening tests (48). Our results also indicated that education was not significantly associated with any of the other outcomes measured at T1 including eHealth literacy. This is again in contrast to previous research in which more education was significantly associated with greater eHealth literacy (52).

There are three theoretical approaches for developing information materials so that they can be used successfully by people of varying degrees of eHealth literacy. One approach is to use tailoring so that a certain version of the information material is adapted to a person's unique characteristics (which are derived from an initial individual assessment of influential factors) (53). Another approach is targeting where information material is intended for a certain subgroup (53). While these two approaches can be applied to all kinds of background variables, specific to health literacy, a third approach has been proposed: the universal precautions approach (54). Information materials are developed in a way so that people can understand them independent of their level of health literacy. This approach is mainly driven by two notions: (1) It is impossible to accurately identify those for whom a certain version is suitable, and (2) health literacy can be situational (e.g., depending on the person's stress level or on the health issue of interest) (54).

When we developed our DA, we intended it to be understandable for all women aged 50, yet we could not be sure whether it would be possible to successfully develop a DA that works equally well regardless of eHealth literacy or education level. Since we did not find a differential effect, it is reasonable to assume that our DA was close enough to a universal precautions approach. This would imply that our DA included sufficient explanatory information sections that can be accessed if desired. Low health literacy individuals are in special need of decision support as they are less likely to be willing to engage in decision making, have higher decision uncertainty, and decision regret (8). Therefore, a DA can be especially valuable for them.

### 4.1. Limitations

The most important limitation of our research was that we used only one instrument to assess eHealth literacy. First, self-reported and objective health literacy show very different associations (46). Also no association between the eHEALS and performance on eHealth tasks was found in previous research (20). This may indicate that there may be a “non-association” between self-reported eHealth literacy (like the eHEALS) and a performance based measure (like our knowledge index). Second, the eHEALS may be a somewhat anachronistic instrument since people today have much more ubiquitous access to the internet than when the eHEALS was created. The eHEALS was developed prior to the proliferation of social media and Web 2.0 technology (41). Some items may thus not be suitable anymore to differentiate between people with high and low eHealth literacy. Third, there is controversy surrounding the factor structure of the eHEALS. The factors we used to assess the effect of eHealth literacy had shown good model fit in our data; nevertheless more research is needed to arrive at a definite factor structure for the eHEALS. Fourth, all subscales had only 2–3 items possibly affecting their reliability. This may be especially severe considering that only the information-use subscale may have assessed relevant aspects of eHealth literacy in this study context. Considering all possible limitations of the eHEALS, it has to be noted that there would not have been a more fitting instrument for this research and using several instruments might have increased the burden of answering an already extensive questionnaire to an unacceptable level. All health literacy measures are somewhat limited in scope and inadequate. The eHEALS is the most used tool for eHealth literacy assessment (19). A systematic review of measures of eHealth literacy found that of 53 articles 45 used the eHEALS to assess eHealth literacy (55). Accordingly, the eHEALS can be regarded as an accepted standard measure (41).

It is questionable in how far our sample was representative regarding eHealth literacy levels. We did not find an interaction between the DA and the eHealth literacy levels of our sample; it is possible that women with extremely low health literacy levels may simply not have participated in the study and therefore a differential effect cannot be ruled out altogether. Another important point is that the results we found in our German-speaking sample with the German translation of the eHEALS may not be applicable to other language versions of the eHEALS. Our sample had a higher education level than the population of women aged 50–54 in North Rhine-Westphalia. Of the women in our study, 45.5% had a university or university of applied sciences entrance qualification compared to 32.9% in the population (56). 41.2% had an intermediate school certificate but only 32.5% of the population have this degree (56). Only 10.4% had obtained a secondary general school certificate compared to 27.8% in the population (56).

### 4.2. Future Research

Future research should assess whether eHealth literacy has an effect on the other dimensions of informed choice (attitude and intention/uptake). In previous research, women with low literacy were more likely to have negative attitudes about mammography (11). One study found that lower literacy women had lower odds of mammography uptake in the past 2 years (57). Additionally, the association between health literacy measures and health outcomes is well-established, but what is less understood is the process linking these two constructs (10). It could be mediated by health actions including uptake of health care (10). Decision making on mammography screening of women with low literacy was also associated with stronger persuasibility by friends and relatives (11). Low health literacy is associated with worse health outcomes (9), but the mechanisms by which this can be explained are less clear (58).

Another important aspect for future research is the circular process through which exposure to information materials and eHealth literacy may influence each other. Being in contact with a credible source of health information on the internet has been shown to be associated with higher eHEALS levels (59). This implies a circular process where people with higher eHealth literacy find better information sources which in turn increases their eHealth literacy.

### 4.3. Conclusion

This research showed that a 3-factor structure of the eHEALS has the most favorable model fit adding to a conflicting picture of the factor structure of the eHEALS. The DA developed in this project increases knowledge irrespective of level of eHealth literacy. This means that not only women with high eHealth literacy profit from the DA but that the DA has been successfully conceptualized as a comprehensible information tool that can be used by women of varying eHealth literacy levels.

## Data Availability

Data are available upon request due to ethical restrictions. Our data contain information that may allow identification of study participants and our consent form did not cover publication of the raw data. For these data protection reasons, data can only be made available to interested researchers without socio- demographic variables or in aggregated form. Interested researchers may submit requests to the Data Protection Officer of Bielefeld University, Ms. Anja Schmid. Contact: Bielefeld University, P.O. Box 10 01 31, D-33501 Bielefeld, Germany. E-mail: datenschutzbeauftragte@uni-bielefeld.de.

## Author Contributions

MR and PK contributed to the conception and design of the study. MR performed the statistical analysis. RS commented on the statistical analysis and model development. MR wrote the first draft of the manuscript. All authors contributed to manuscript revision, read and approved the final version.

### Conflict of Interest Statement

The authors declare that the research was conducted in the absence of any commercial or financial relationships that could be construed as a potential conflict of interest.
